# Debates in Allergy Medicine: Allergy skin testing cannot be replaced by molecular diagnosis in the near future

**DOI:** 10.1186/s40413-017-0164-1

**Published:** 2017-09-19

**Authors:** Désirée Larenas-Linnemann, Jorge A. Luna-Pech, Ralph Mösges

**Affiliations:** 1 Investigational Unit, Hospital Médica Sur, Torre 2, consultorio 602, Puente de Piedra 150, Col. Toriello Guerra, Del. Tlalpan, 14050 Mexico City, Mexico; 2 Departamento de Disciplinas Filosófico, Metodológico e Instrumentales, CUCS, Universidad de Guadalajara, Guadalajara, Mexico; 3 Institute of Medical Statistics and Epidemiology, Faculty of Medicine, University of Cologne, Cologne, Germany

**Keywords:** Allergens, Skin testing, Prick testing, Specific IgE, Molecular allergy diagnostics, Peanut, Oral allergy syndrome

## Abstract

Percutaneous skin prick tests (SPT) have been considered the preferred method for confirming IgE-mediated sensitization. This reliable and minimally invasive technique correlates with in vivo challenges, has good reproducibility, is easily quantified, and allows analyzing multiple allergens simultaneously. Potent extracts and a proficient tester improve its accuracy.

Molecular-based allergy diagnostics (MA-Dx) quantifies allergenic components obtained either from purification of natural sources or recombinant technology to identify the patient’s reactivity to those specific allergenic protein components. For a correct allergy diagnosis, the patient selection is crucial. MA-Dx has been shown to have a high specificity, however, as MA-Dx testing can be ordered by any physician, the pre-selection of patients might not always be optimal, reducing test specificity. Also, MA-Dx is less sensitive than in vitro testing with the whole allergen or SPT. Secondly, no allergen-specific immunotherapy (AIT) trial has yet shown efficacy with patients selected on the basis of their MA-Dx results. Thirdly, why would we need molecular diagnosis, as no molecular treatment can yet be offered? Then there are the practical arguments of costs (SPT highly cost-efficient), test availability for MA-Dx still lacking in wide areas of the world and scarce in others. As such, it is hard physicians can build confidence in the test and their interpretation of the MA-Dx results. In conclusion: as of now these techniques should be reserved for situations of complex allergies and polysensitization; in the future MA-Dx might help to reduce the number of allergens for AIT, but trials are needed to prove this concept.

## Background

The increased prevalence of allergic diseases makes it mandatory to use quick, precise, and reliable diagnostic tools. To make the diagnosis of a specific allergy, several components are needed: a subject with symptoms corresponding to an allergic disease, a physician knowledgeable of allergic disorders and specific allergy tests, the availability of quality allergy testing instruments—in vitro and/or in vivo—and finally, and perhaps most importantly, a physician capable of interpreting the test results in light of the patient’s symptoms. Only if all of the above components are “checked off the list” is it very likely for a correct allergy diagnosis to be made. In this article, our objective is to discuss the part of “allergy testing,” but right from the start it can already be assumed that a discussion of allergy testing is more useful, proactive, and better assessed when the complete context of allergy diagnosis is considered. It all starts with a patient presenting with symptoms and signs suggestive of allergic diseases, particularly allergic rhinitis (with or without allergic conjunctivitis), allergic asthma, food allergy, or even anaphylaxis. A positive personal and family history of allergic diseases, together with a clinical history of fluctuating symptoms over time, sometimes within the course of a day, or even within the course of a year, makes the diagnosis of allergy more plausible. An exacerbation of the symptoms following exposure to triggers could add further clinical support to the suspicion that we are dealing with an allergy, mainly if symptoms exacerbate on exposure to a certain potential allergen (e.g., cat, dog, horse, house dust mite) or year after year during the same months (pollen season). However, determining which of the patient’s allergen(s) might be based on the clinical history only is not considered adequate, as clinical observations are subject to a high degree of error; [1] hence the relevance of having accurate and confident specific allergy testing available [2] (Tables 1 and 2).

**Table 1:** Characteristics of various specific allergen tests

Test	Substance tested	Number of allergens tested per test	Readout
Allergy tests in vivo			
● Skin prick test	sIgE to whole natural allergen	On average 40 allergens	Semi-objective (physician measures wheal/flare)
● Nasal provocation test	sIgE to whole natural allergen	1 allergen at a time (maximum 3–4 per session)	Subjective/Objective
● Conjunctival provocation test	sIgE to whole natural allergen	1 allergen at a time (maximum 3–4 per session)	Subjective/Objective
Allergy tests in vitro			
● sIgE to a batch of allergens (Immulite, Microtest, RAST)	sIgE to whole natural allergen (s)	On average 20–60 allergens	Objective
● Molecular-based allergy diagnostics: sIgE to microarray-based allergen protein components (ImmunoCAP [single allergen assay], ISAC [multiplex assay])	sIgE to allergen protein components	1 to >100 allergen protein components	Objective
● Basophil/histamine release, BAT	Effect of allergen on patient’s basophils	1 allergen at a time (maximum several allergens/session)	Objective

*sIgE* specific IgE, *ISAC* immuno solid-phase allergen chip, *RAST* radioallergosorbent test, *BAT* Basophil activation test

**Table 2:** Comparison of some advantages and limitations of skin prick test and molecular-based allergy diagnostics for allergy confirmation (adapted from 3,5)

Skin tests (extract based)	Molecular-based sIgE tests (component based)
Available only where equipment, reagents and trained staff are on hand.	Available only in laboratories with high-end machinery where specific reactives and trained staff are on hand.
Moderately costly.	High cost.
Minor discomfort for scratching, itch if positive.	Minor pain. Venesection may be painful or anxiety-provoking (particularly in children),
Requires patient cooperation. Performance in small children may be limited.	Little patient effort or cooperation required.
Slight risk of systemic allergic reaction (more so in some special situations). A convincing recent history of anaphylaxis represents a contra-indication.	No risk to patient; may be first line with certain high-risk allergens.
Require areas of normal skin for testing.	Can be done regardless of extensive skin disease.
Must stop antihistamines and some other drugs several days before test.	Can be done regardless of taken medications.
Methodology and result quality variable, standardization not always possible. No formal quality control at the current time.	Laboratory test subject to strict quality control, reagent availability and technique standardization.
Results in 30 min	Results may take days/weeks.
Results are visible and compelling to patients; may have value in ensuring compliance with allergen avoidance measures.	Results are not directly meaningful to patients.
Can extemporaneously prepare allergens (with appropriate considerations; specialist practice).	Some food allergens, drugs and pollens not available for testing.
In most cases have better sensitivity for clinically relevant allergies.	Reasonably good sensitivity.
Fresh food allergens (prick-to-prick) available with good sensitivity.	Fresh allergens not available.
No interference from high total IgE.	False positives possible with high total IgE levels.
Numerical measurements may vary by different operators.	Numerical results obtained on different types of equipment are not directly comparable.

Table 1 summarizes in a non-exhaustive way some characteristics of specific allergen tests to help the reader differentiate between the methods used and some practical details of each one of the tests. Molecular-based allergy diagnostics (MA-Dx) is a variant for determining specific IgE (sIgE) in serum (or any other body fluid tested) that quantifies allergenic components obtained either from the purification of natural sources or recombinant technology in order to identify the patient’s reactivity to specific allergenic proteins (rather than the whole allergen). As such, MA-Dx is able to discriminate between allergy to the major allergen from house dust mite Der p 1, or Der p 2 or Der p 21, for example, as opposed to the traditional IgE testing (in vivo or in vitro) that typically reports positivity to *Dermatophagoides pteronyssinus* in general.

Two modalities of the microarray technique are commonly recognized: ImmunoCAP, which uses panels of single allergens together with the corresponding allergen extract, and Immuno-Solid phase Allergen Chip (ISAC), which enables testing for specific IgE against multiple allergen components in a multiplex assay [3, 4]. Although MA-Dx undoubtedly constitutes a promising tool in allergy diagnosis, its current use in clinical practice is still highly selective and only considered as a complementary diagnostic test, when a detailed clinical history and traditional extract-based IgE tests (such as SPT or in vitro sIgE tests) are inconclusive or contraindicated.

In this review, we shall discuss several evidence-based and practical arguments to establish that, in most cases, conventional in vivo methods to confirm allergy sensitivity (such as SPT) should not currently, nor in the near future, be replaced by MA-Dx. However, they could be very useful as a complementary diagnostic modality in selected cases. For practical reasons, other skin testing modalities (i.e., intracutaneaous tests) or older in vitro sIgE techniques (i.e., RAST) are not included in this debate.

## Arguments

As diagnostic reliability to confirm allergic sensitization is mandatory, it is very important to emphasize that these tests should always be considered as complements to the prime diagnostic tool: a careful medical history and physical examination. Moreover, both, SPT and MA-Dx require skill and knowledge for a correct interpretation of results, [5] and accurate application to the clinical entity of the patient. Both exhibit diagnostic advantages and limitations (Table 2). Although promising, MA-Dx is not currently substituting traditional SPT, and in most cases is considered as a third-line approach, after the clinical history and SPT or sIgE testings, as has been clearly stated on evidence-based consensus such as the WAO-ARIA-GA2LEN consensus on MA-Dx [3] This can be sustained considering several scientific and practical arguments.

### Scientific arguments

Outside the context of clinical trials and medical research, tests should only be run in day-to-day medical practice if their results lead to a certain action. In allergy in particular, testing is carried out with a triple objective: to confirm the diagnosis of allergy (A); to suggest specific avoidance measures to the patient (B); and to guide the preparation of specific-allergen immunotherapy (AIT) (C).

We shall argue below how these three objectives are better met by classic allergy testing, as compared with MA-Dx.

#### Determination of sIgE in vivo to the whole allergen is more sensitive than MA-Dx

To date, very few studies have compared the accuracy of MA-Dx to traditional in vivo tests in allergic patients, mainly in the context of food allergy and with an oral food challenge as the reference standard. In general, MA-Dx tended to have higher specificity, but lower sensitivity relative to the extract-based whole allergen SPT for the prediction of allergic response, but the diagnostic performance of the in vitro tests varied largely between studies, depending on the allergens investigated and the way in which MA-Dx testing was used.

Ott, et al. [6] compared the accuracy of ISAC containing eight individual components (α, β and κ casein, Bos d4, Bos d5, Gal d1, Gal d2, Gal d4) with the accuracy of SPT (native hen’s egg or native cow’s milk). SPT had the highest sensitivity for cow’s milk allergy, 93.6% (95% CI: 78.5–99%), whilst all five ISAC components assessed had low sensitivity for cow’s milk allergy (range: 23.9–50%). On the contrary, all five ISAC 51 components had high specificity for cow’s milk allergy (range 88.4–97.7%), whereas SPT had low specificity, 48.2% (95% CI: 28.7–68%). Similarly, Alessandri et al. [7] assessed allergy to raw and boiled egg, concluding that SPT had the highest sensitivity for predicting allergic response to raw egg white, 88% (95% CI: 71.8–96.6%), while Gal d3 measured using ISAC had the highest specificity, 100% (95% CI: 90–100%). Results using boiled egg were very similar to raw egg for both testing modalities. Perhaps the more promising results of MA-Dx in the field of food allergy come from peanut allergy, by recognizing sIgE antibodies to Ara h2 as the most common peanut allergen associated with clinical reactivity, and that sensitization to Ara h1, 2, or 3 has been related with more severe clinical reactions in some subjects [8]. However, studies in this matter have shown several limitations and inconsistencies, as has been pointed out in the most recent AAAAI/ACAAI/JCAAI food allergy position paper [9]. For hazelnut allergy, Albarini et al. [10] compared four components measured by ISAC (Cor a1 1010, Cor a1 0401, Cor a8 and Cor a9) to SPT, which had 100% sensitivity, while the ISAC components had low sensitivity (range: 6.3–56.3%). In this study, the ISAC components had higher specificity (range: 73.7–100%) than SPT (52.6%).

As far as we can ascertain, only two comparative studies investigating the accuracy of MA-Dx for aeroallergen mediated allergy have been published [11, 12], and both used SPT as the reference standard. Conversely, De Swert et al. [13] investigated soy flour allergy, comparing the measurement of the soy flour component rGly m4 by using ISAC to serum IgE to the same component and to SPT for soy flour. ISAC reported the highest sensitivity, 86% (95% CI: 42–100%), but also the lowest specificity, 80% (95% CI: 28–100%). Single sIgE ImmunoCAP testing and SPT had similar sensitivity (75%) and specificity (100%).

All aforementioned studies investigated the diagnostic performance of a relatively limited range of MA-Dx components of a specific allergen. Thus, these studies are somehow unable to provide any information on the sensitivity/specificity of the whole allergen panel. We consider this shortcoming to be a serious limitation, because, for example, it remains unclear to what degree MA-Dx testing may produce false-positive results by detecting sensitizations, which are not always clinically relevant.

Some evidence suggests that MA-Dx can be useful for distinguishing between structurally similar allergens that cross-react with the same IgE antibody [3]. This knowledge can be used to specifically avoid contact with the causative allergen in food allergy and idiopathic anaphylaxis, but its use also has been associated with large numbers of clinically false–positive test results. For example, a study from the UK [14] showed that the addition of ImmunoCAP and ISAC to standard diagnostic work-up could identify a potentially causative allergen in previously undiagnosed patients. At the same time, however, using MA-Dx also resulted in the identification of a large amount of sensitizations that were not considered to be clinically associated with the anaphylaxis. Thus, MA-Dx results still need to be taken with caution in order to limit potentially unnecessary allergen avoidance strategies.

#### Preparation of AIT based on skin test vs. MA-Dx results

The selection of the allergen(s) for use in AIT has historically been based on the results of skin testing. Until today, no clinical trial has shown the efficacy of AIT by selecting the patients and allergens solely on the basis of MA-Dx test results. Much less has it been shown that selecting allergen(s) for AIT based on MA-Dx could lead to a more efficient or safer AIT, as opposed to AIT with allergen(s) selected according to SPT results.

In vitro diagnosis, when combined with a positive SPT in selecting patients for grass pollen sublingual AIT with tablets resulted in enhanced clinical efficacy in one study [15]. Again, the primary patient selection criterion for inclusion in this trial was the SPT. The determination of the exact allergen(s) for AIT can be facilitated using a secondary test, but the preferable option should be an end-organ challenge test: nasal or conjunctival challenge testing is used by many allergists in Europe to reduce the number of allergens for AIT to one or very few [16].

Some published evidence tends to favor MA-Dx as a more adequate tool than traditional skin testing to decide which allergens to use in AIT [17], but in general their results could not be considered fully definitive. In a trial involving 141 patients with respiratory allergy in Spain, Sastre et al. [18] showed that the number of allergens to be applied in AIT could be reduced considerably or modified when using MA-Dx (with disagreements on AIT prescription when ImmunoCAP results were assessed vs. SPT up to 79 [54%] of cases), implying that this molecular approach can be considered more accurate than the in vivo test. However, in this study’s result, no details were given regarding which specific AIT prescriptions were actually used. Moreover, they based their results by measuring interobserver agreement, which is an approach we consider highly prone to suffering subjective biases. More importantly, the authors did not go on to show a hypothetical higher efficacy in the sense of symptoms or medication reduction of such an MA-Dx-based AIT.

Published evidence favoring MA-Dx has been found to have more value regarding *Hymenoptera* venom allergens, where the selection of the correct allergens for venom immunotherapy (VIT) has proven to be truly enhanced by molecular diagnosis. Furthermore, in vivo sting testing can potentially induce systemic reactions, but even in this subgroup of allergic patients, the benefit of MA-Dx applies exclusively to some very selected cases of multiple venom allergen positivity, or to those with a history of an adverse reaction to a *Hymenoptera* sting with negative SPT results [19]. Regardless of all these considerations, the only currently recommended diagnostic strategy for predicting the success of VIT is the sting challenge with a living insect [20]. Since these sting challenge tests can induce severe systemic reactions, in vitro methods for predicting the success of VIT would be preferable, but the evidence supporting this strategy is still limited.

#### Molecular diagnosis without molecular treatment

AIT is done with extracts of whole allergens. Some groups have investigated AIT with (modified) peptides for cat [21], birch [22], or a mix of several house dust mite molecular allergens in one report to date [23], but these treatments are still considered experimental. Moreover, molecular treatment has still not been developed for most allergens. MA-Dx is said to be more accurate, and thus could represent a better guide for determining which specific allergens should be selected for AIT administration [3, 17], but some very recent evidence strongly recommends single allergen AIT in polyallergic patients in whom one of the relevant allergens is clearly responsible for the symptoms [24]. It seems perfectly plausible to achieve this based on SPT results alone. If this is the preferred practice, the reality of patient tailored AIT exclusively based on MA-Dx indeed seems still a distant prospect.

#### Absence of natural adjuvants in molecular AIT

There might be another (albeit hypothetical) argument against purified molecular AIT. The efficacy of AIT can be enhanced by some adjuvants, for example, some toll-like receptor (TLR) ligands, such as lipopolysaccharides [25]. Some natural allergens have been shown to contain TLR stimulating capacity [26], and this important potential effect would be lost if only a certain protein or protein component were used for molecular AIT.

### Practical arguments

#### The cost of MA-Dx is too high

When considering test costs, service and maintenance costs, and personnel costs for performing and interpreting the results, it is easy to recognize that MA-Dx tests are onerous, and can carry a substantial financial burden for laboratories, patients and/or insurance companies. As a clear example, a recent UK-based comparative cost analysis [27] reports a per person cost of £219.51 for an ISAC microarray panel (using a LuxScan 10 k reader, allowing 4 allergens per kit), £136.37 for sIgE testing (on average 8 allergens measured per patient), and £62.28 for SPT, respectively. In the US, the cost of a complete 112 microarray-based allergen molecule ISAC panel is about $300 [28]. In Latin-American countries this is about 600 USD, 8.25 times the Mexican minimum monthly salary, and more than ten times the cost of a 30 allergen SPT.

#### Limited availability of MA-Dx tests

A very practical argument is that, in many parts of the world, MA-Dx tests for allergy are not yet available, neither the necessary laboratory equipment nor the trained personnel to adequately run the kits, which greatly limits the possibility for allergy care physicians to gain experience with such diagnostic techniques. To run microarray plates for MA-Dx, a special microchip reader machine is needed, and trained personnel capable of managing and maintaining it is mandatory. The reagents and consumables usually have to be imported, raising the maintenance costs. Consequently, many laboratories are reluctant to venture into the MA-Dx territory, as the cost-benefit ratio can only be balanced to the benefit side when enough tests are run.

#### Physicians confidence in the test: correct interpretation

As we set out to emphasize from the start: the final step in allergy diagnosis is the correct interpretation of the test results. Thus, to get MA-Dx well established as a routine diagnostic tool, physicians need to become acquainted with it and be able to gain confidence in the correct interpretation of the results. Microarray analyses are also prone to limitations and errors due to imprecise technical material and imperfections in the technique for hybridization and scanning (i.e., deviations in the amount of biologic material printed in each microarray spot, variations in the amount of the fluorescent reactive used to mark samples, errors inherent to the light measurement by the scanner, among others), in addition to the inherent difficulties, related to gene material stability and its processing per se. In many parts of the world, allergists do not feel comfortable (yet) interpreting MA-Dx results and are even less acquainted with how to put these results into practice. As long as only a few allergists use these tests for a very limited number of their patients, it does not seem that this apparent lack of confidence is going to change in the near future.

## Conclusion

Even though MA-Dx technology constitutes an innovative and promising area, such techniques should be considered a complementary, more selective, third-line diagnostic modality reserved for very specific cases, such as complex allergies and polysensitization. Furthermore, it should be regarded as an add-on diagnostic approach that might help to identify homologous allergens that by their cross-reactivity might explain the clinical symptoms of oral allergy syndrome linked to respiratory allergy to pollen, and as a tool to predict the risk for more severe adverse food allergy reactions (i.e., Ara h 2 versus Ara h 8 positivity).

In the future, it is probable that MA-Dx will help to reduce the number of allergens to be administered in AIT, but efficacy data in this regard are still absent. Moreover, no molecular tools are available to date, which allow the prediction of AIT outcomes. The cost-benefit is another very important problem regarding MA-Dx. In countries with a low gross domestic product, the decision to recommend an expensive test, such as MA-Dx, should be made carefully and, again, limited to very specific cases. In more wealthy countries or communities, issues such as access and insurance coverage would be important to consider.

We can conclude that, until new and better-designed investigations provide more solid evidence in this regard, MA-Dx shall not completely replace traditional SPT or challenge tests as the first-line approach to confirm specific allergy at present nor in the near future. Nevertheless, performing both in vitro and in vivo tests may undoubtedly contribute to improve sensitivity/specificity and the overall allergic diagnostic accuracy under specific circumstances.

## Abbreviations

AIT: Allergen-specific immunotherapy
CI: Confidence interval
IgE: Immunoglobulin E
ISAC: Immuno solid-phase allergen chip
MA-Dx: Molecular-based allergy diagnostics
RAST: Radioallergosorbent test
sIgE: Specific immunoglobulin E
SPT: Skin prick test
TLR: Toll-like receptor
UK: United Kingdom
US: United States of America
USD: United States dollars
VIT: Venom immunotherapy
